# Early enteral nutrition versus delayed enteral nutrition in patients with gastrointestinal bleeding: A PRISMA-compliant meta-analysis

**DOI:** 10.1097/MD.0000000000014864

**Published:** 2019-03-15

**Authors:** Hongyan Zhang, Yu Wang, Shujun Sun, Xin Huang, Guangjie Tu, Jingxu Wang, Yun Lin, Haifa Xia, Yin Yuan, Shanglong Yao

**Affiliations:** a Department of Cardiology; b Department of Anesthesia; c Department of Critical Care Medicine, Institute of Anesthesia and Critical Care, Union Hospital, Tongji Medical College, Huazhong University of Science and Technology, Wuhan, China.

**Keywords:** enteral nutrition, gastrointestinal bleeding, meta-analysis, systematic review

## Abstract

**Background:**

Controversy persists about whether early enteral nutrition administration is related to worse prognosis than delayed enteral nutrition for patients with gastrointestinal bleeding.

**Objectives:**

To systematically evaluate the effect of early enteral nutrition on the patient with gastrointestinal bleeding through the meta-analysis.

**Methods:**

Such electronic databases including PubMed, EMBASE, Cochrane Library, CNKI, and CBM were searched from 1985 to March 2018. Randomized controlled trials that compared early enteral nutrition versus delayed enteral nutrition in patients with gastrointestinal bleeding were considered eligible. Data extraction and the methodological quality assessment of the included trials were carried out according to the Cochrane Handbook. We calculated the pooled risk ratio, weighted mean difference, and the corresponding 95% confidential interval using RevMan5.3.

**Result:**

A total of 5 trials involving 313 patients were included. Compared with delayed enteral nutrition, there was a tendency for a decreased rebleeding rate in the early enteral nutrition group, but the trend was not statistically significant (risk ratio = 0.75, 95% confidential interval: 0.34–1.64, *I*^2^ = 0). As for mortality within 30 days, no significant difference was found between the 2 groups (risk ratio = 0.74, 95% confidential interval: 0.23–2.39, *I*^2^ = 0). In addition, the pooled analysis showed that early enteral nutrition was related to reduced hospitalized days (weighted mean difference = −1.69, 95% confidential interval: −2.15 to −1.23; *I*^2^ = 27%)

**Conclusion:**

For patients with gastrointestinal bleeding, early enteral nutrition within 24 hours does not result in the significantly higher risk of rebleeding and mortality compared with delayed enteral nutrition, but decrease hospitalized days. Patients who are at low risk for rebleeding can be fed early and discharged early. However, larger, high-quality randomized controlled trials are needed to verify these findings, and when the gastrointestinal bleeding patient start enteral nutrition is worth studying.

## 1 Introduction

Gastrointestinal bleeding (GIB) is a severe complication of a variety of diseases like the chronic liver disease that result in the esophageal variceal bleeding, peptic ulcer on account of excessive gastric acid secretion, *Helicobacter pylori* infection, stress ulcer owing to shock, trauma, postoperative or severe systemic infection, and so on. It is one of the most common gastrointestinal emergencies, with an average mortality rate of 10% in a multicenter study conducted in all UK hospitals.^[1]^ Despite advances in the diagnosis and management of GIB, the mortality rate has not changed significantly in the last 50 years.^[1,2]^ Upper GIB from peptic ulcers or other nonvariceal causes generally stops spontaneously, if not, aggressive management is required. Such measures are also necessary for patients at high risk for rebleeding.^[3]^ Although its treatment has evolved rapidly in recent years, the prognosis remains poor with further bleeding or rebleeding. To improve the prognosis, combination therapy is crucial. In the multidisciplinary care of patients, nutritional support has become a relevant strategy.^[3–5]^ As for nutrition therapy on patients with GIB, it is customary for clinicians to institute absolute fasting for 48 to 72 hours. Fasting is believed to improve the ability to control intragastric pH, stabilize clots, and reduce the risk of rebleeding.^[6]^ Enteral feedings are usually withheld for 72 hours in GIB patients because the possibility of rebleeding is significantly higher in the first 72 hours, and fasting may reduce gastric secretion and gastric inflammation.^[7]^ However, studies have shown no difference in intragastric Ozawa et al studied 49 *H. pylori*-positive patients with bleeding gastric ulcers. The results showed no significant differences in intragastric pH of patients receiving acid-reducing medications (both ranitidine and omeprazole) among fasting groups and early fed groups.^[8]^ And several randomized controlled trials (RCTs) showed that early enteral nutrition (EEN) had no significant effects on treatment outcomes in patients with GIB who were treated with endoscopic hemostasis.^[9–13]^ And it is significant to determine when to start enteral nutrition because early feeding may reduce the cost of treatment and shorten the length of hospital stay.^[9–12]^ In the course of fasting, parenteral nutrition may be used. If patients fed through a catheter by total parenteral nutrition, there is the possibility of vascular catheter-site infection which causes septicemia and thrombophlebitis. Parenteral nutrition may have disadvantages that the empty gastrointestinal tract may lose its integrity and barrier function.^[9]^ Parenteral nutrition may promote bacterial translocation from the gut by increasing the cecal bacterial count and impairing intestinal defense.^[14]^ The risk of rebleeding depends on the etiology and the severity of diseases. A fairly large number of patients are classified as low risk for rebleeding and can be safely fed immediately or the same day and discharged early. In case of the ulcer with low risk of rebleeding (Forrest II c and III) or in patients with gastritis, Mallory–Weiss, oesophagitis, or angiodysplasia, there is no need to delay refeeding, and they can be fed as soon as tolerated.^[6]^ The impact of early feeding after treatment of GIB has rarely been well investigated, probably for fear that the nasogastric or nasojejunal tube worsens bleeding. There are a few reviews qualitatively summarized the evidence but no meta-analysis study the prognosis of EEN versus delayed enteral nutrition (DEN) on GIB patients.

Our objective was to perform a meta-analysis for EEN in GIB patients, focusing on specific prognosis indicator compared with DEN.

## 2 Methods

We conducted this study according to the methods in the Cochrane Handbook for Systematic Reviews of Interventions. The findings were reported following the indications of preferred reporting items for systematic reviews and meta-analyses statement.^[15]^ Ethics approval was not required, as our research does not involve patient's personal information and only aggregated nonidentifiable data.

RCTs that compared EEN versus DEN on patients with GIB were considered eligible. Data extraction and quality evaluation of literature were carried out according to the Cochrane Systematic Reviews evaluation method. We calculated the pooled risk ratio (RR), weighted mean difference (MD) and the corresponding 95% confidential interval (95% CI) using RevMan5.3. The primary outcome was the rebleeding rate who had GIB and received EEN. The secondary outcomes were mortality and hospitalize days.

### 2.1 Literature search

Two investigators independently conducted an electronic literature search for relevant studies concerning EEN in PubMed, EMBASE, Cochrane Library from 1985 to March 2018, using keywords and subject term. Additionally, we searched the Chinese Biomedical Literature Database concluded CNKI and CBM (1985 to March 2018). During the search, no language limits were set. We also searched the ClinicalTrials.gov registry in 2018 to identify additional clinical trials. The following terms and strategies were used to search the databases: “enteral nutrition OR enteral feeding OR feeding” and “gastrointestinal bleeding OR gastrointestinal hemorrhage OR intestinal bleeding OR intestinal hemorrhage OR bleeding.” We also scrutinized the citation lists of relevant meta-analyses and reviews to avoid missing qualified trials.

### 2.2 Study selection

RCTs were included in the following steps. First, we screen the identified records through database searches by title and abstract. The primary inclusion criteria and exclusion criteria are as follows.

#### 2.2.1 Studies included in this meta-analysis had to fulfill the following criteria

(1) We included RCTs addressed the prognosis of EEN compared to DEN on patients with GIB;
(2) The definition of EEN and DEN are basically consistent in each literature;
(3) The treatment group was treated with EEN, and the control group was treated with parenteral nutrition or DEN;
(4) The outcome measures of each literature are basically the same;
(5) The baseline characteristics of the literature are comparable.

#### 2.2.2 Studies were excluded if

(1) The articles are not RCT;
(2) The articles are of poor quality or lack enough information;
(3) The articles are not related to the prognosis of the treatment.

Then we conducted a full-text articles assessment for eligibility. We formulated a specific standard through full-text reading to screen the study, as regards the types of studies, types of participants, types of interventions and types of outcome measures. The detailed standards are:

(1) types of studies: RCT;
(2) types of participants: the patient with GIB;
(3) types of interventions: the treatment group was treated with EEN within 24 hours after the treatment of GIB, and the control group was treated with parenteral nutrition or DEN (beyond 24 hours);
(4) outcome measures: 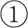 the primary outcome is rebleeding rate, the secondary outcomes are 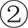 mortality rate and 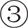 hospitalization days.

Full-text screening form is shown in the following Table 1

**Table 1:**
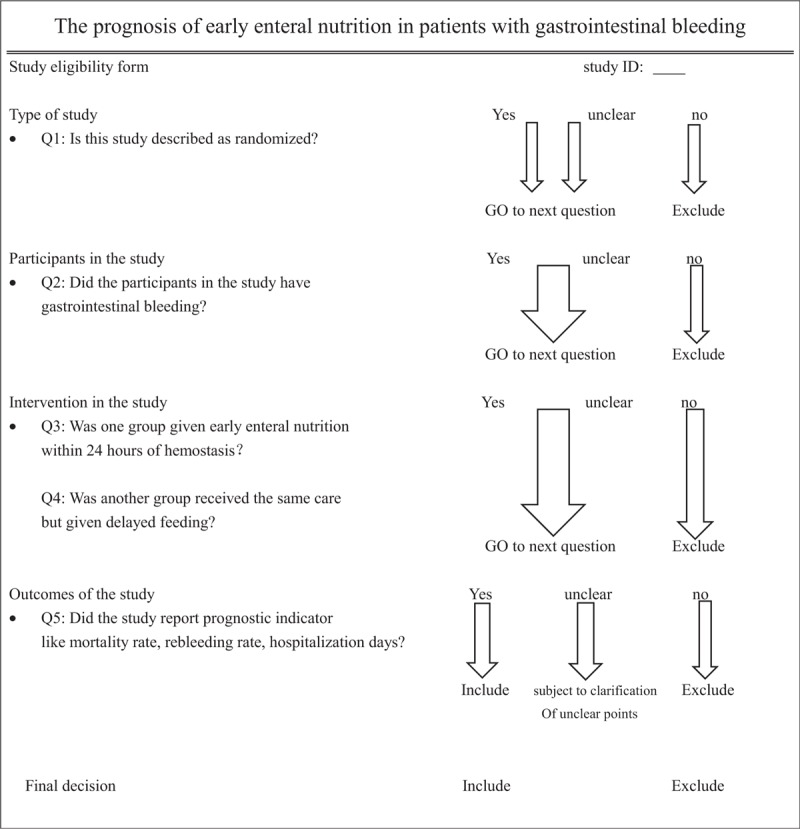
Full-text screening form.

### 2.3 Data extraction

Two of us (WY and HYZ) used a standardized spreadsheet to extract data independently. Disagreements were resolved by consensus after contact with the senior author. We extracted the following study characteristics and information:

(1) First author, publication year, number of participants;
(2) Study patient characteristics like country, gender, age, and the primary pathogenesis of GIB;
(3) Protocols of nutrition therapies like initial time, nutritional ways, and nutrients, the hemostatic treatment;
(4) The primary and secondary outcomes.

### 2.4 Quality assessment and bias assessment

We assessed the risk of bias for each trial using the Cochrane risk-of-bias tool.^[16]^ Accordingly, the following issues were evaluated:

(1) random sequence generation;
(2) allocation concealment;
(3) blinding of participants and personnel;
(4) blinding of outcome assessment;
(5) incomplete outcome data adequately addressed;
(6) free of selective reporting;
(7) free of other bias.

The internal validity criteria that refer to characteristics of the study that might be related to selection bias, performance bias, attrition bias, and detection bias. The internal validity criteria should be used to define methodological quality in the meta-analysis.

Risk of bias was independently graded by 2 of us (WY and HYZ) as follows: low risk, high risk, and unclear risk. Any discrepancies between raters were resolved through consensus. Finally, authors of included articles were contacted to obtain additional information on unclear reporting.

### 2.5 Data quantitative synthesis

All analyses were performed using RevMan5.3, establishing the level of significance at a 2-tailed *P*-value < .05. Data of binary outcomes extracted from original RCTs were pooled to estimate the RRs and corresponding 95% CIs. For continuous outcomes, data were pooled to estimate weighted MDs and corresponding 95% CIs. The Mantel–Haenszel *χ*^2^ test and the *I*^2^ statistic were used to measure statistical heterogeneity among the included studies. We considered heterogeneity to be substantial if the *I*^2^ value was 50% or greater or the *P*-value was .1 or less.^[17]^ A random-effect model was used for statistics with noted heterogeneity, otherwise, a fixed-effect model was applied.

## 3 Results

### 3.1 Search results and study characteristics

**Figure 1:**
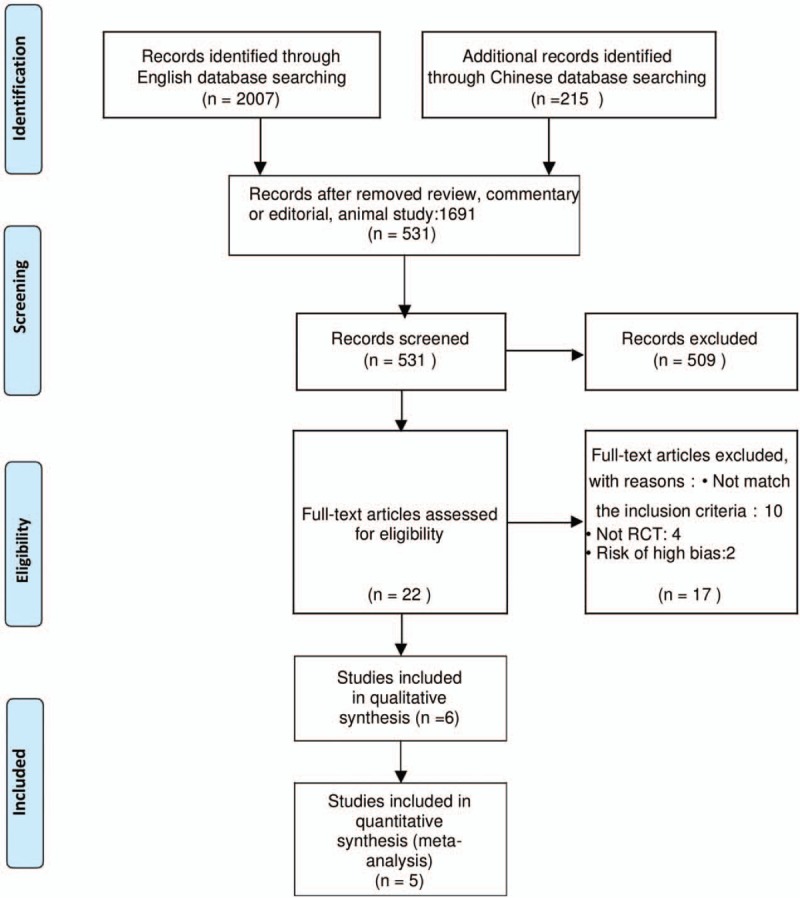
The flow diagram for searching and filtrating of eligible studies.

We identified 2222 articles through the literature search. After excluding 1588 papers through title and abstract review, 22 full-text articles were examined;

Finally, 5 RCTs^[9–13]^ enrolled 313 patients met our inclusion criteria and were included in the meta-analysis. The flow diagram for searching and filtrating of eligible studies is illustrated in Figure 1.

The included trails are expressed by the publication year and the authors, like Hepwort et al. We aggregated the available data on country, age, gender, number of patients, etiology, nutrition method, and hemostatic treatment in Table 2. The included studies are basically similar in the baseline characteristics except for 1 RCT.^[11]^ The number of patients with Forrest Ib and IIa was a little higher in the group B than in the group A and antiacid treatment was not homogeneous. The nutrients were not exactly the same, but all of the nutrients were mixed warm liquid feeding contained enough calories and protein, like soup, milk, or rice in liquid form. Hemostatic treatments of the 5 trails are endoscopic treatment like emergency sclerotherapy, banding ligation, or endoscopic injection therapy with adrenaline. Basically, our results showed no differences between the EEN and DEN groups in terms of these baseline characteristics.

**Table 2:**
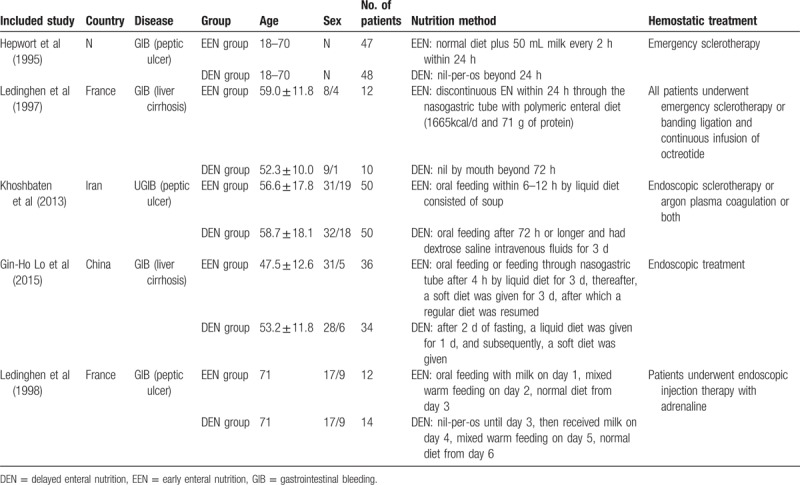
The baseline characteristics.

### 3.2 Included trials quality assessment

The included RCTs was evaluated for quality according to the Cochrane Handbook for Systematic Reviews of Interventions.^[16]^

None of the trials were double-blinded because it was difficult for patients and clinicians to hide the study group. One RCT^[11]^ had other bias on account of differences in baseline characteristics. Patients with Forrest Ib and IIa were more in the group B than in the group A and some patients received ranitidine, while others received proton pump inhibitors. The 5 included studies were well-designed RCTs and substantially of good quality. The assessment is displayed in Table 3.

**Table 3:**
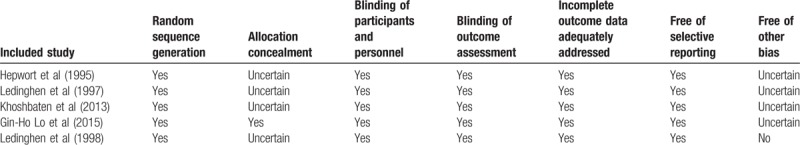
The quality assessment of included studies.

### 3.3 Study outcomes

We calculated the pooled RR, weighted MD, and the corresponding 95% CIs using RevMan5.3. We used a fixed effect model for the heterogeneity of each outcome is less than 50%.

#### 3.3.1 Effect of EEN on rebleeding

**Figure 2:**
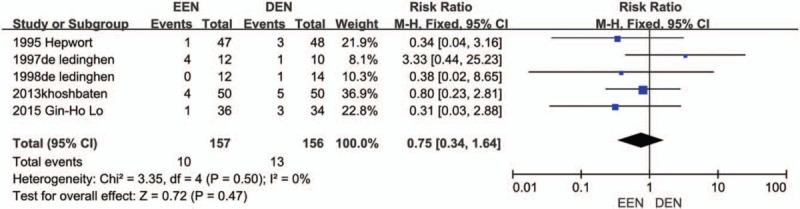
Rebleeding forest plot.

For all the RCTs fulfilling inclusion criteria for quantitative synthesis, it was possible to collect data on the outcomes considered. In the primary analysis, based on all 5 trials,^[9–13]^ the EEN was no associated with rebleeding compared with DEN. Besides, there was a tendency for a decreased rebleeding rate in the EEN group, but the trend was not significant (RR = 0.75, 95% CI: 0.34–1.64, *I*^2^ = 0). The enrolled participants of 313 patients are not enough to verify the finding, however, it makes sense to some extent. Forest plot of the rebleeding rate is displayed in Figure 2.

#### 3.3.2 Effect of EEN on mortality and hospitalize days

**Figure 3:**
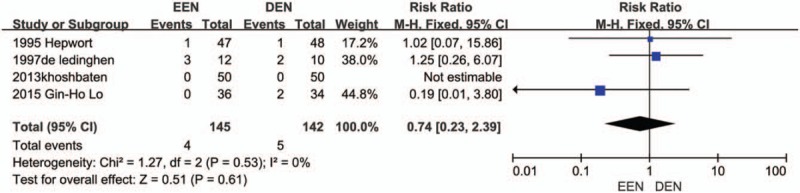
Mortality forest plot.

**Figure 4:**
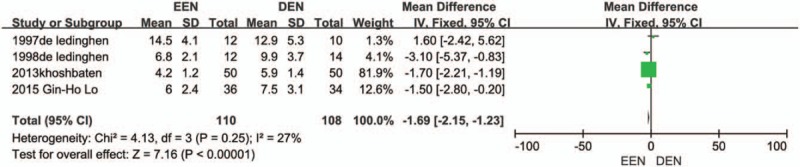
Hospitalized days forest plot.

Four trials^[9,10,12,13]^ presented data on the incidence of mortality of EEN and DEN. There was no significant difference was found between the EEN and DEN groups (RR = 0.74, 95% CI: 0.23–2.39, *I*^2^ = 0). Forest plot of the rebleeding rate is displayed in Figure 3. As for hospitalized days, 4 trails^[9–12]^ reported the data and 3 of them showed a significant reduction of hospital stay in the EEN group versus the DEN group. We aggregated the available data on the hospital stay. The pooled analysis showed that EEN was related to reduced hospitalized days (MD = −1.69, 95% CI: −2.15 to −1.23; *I*^2^ = 27%). Forest plot of the hospitalized days is shown in Figure 4.

#### 3.3.3 Publication bias

**Figure 5:**
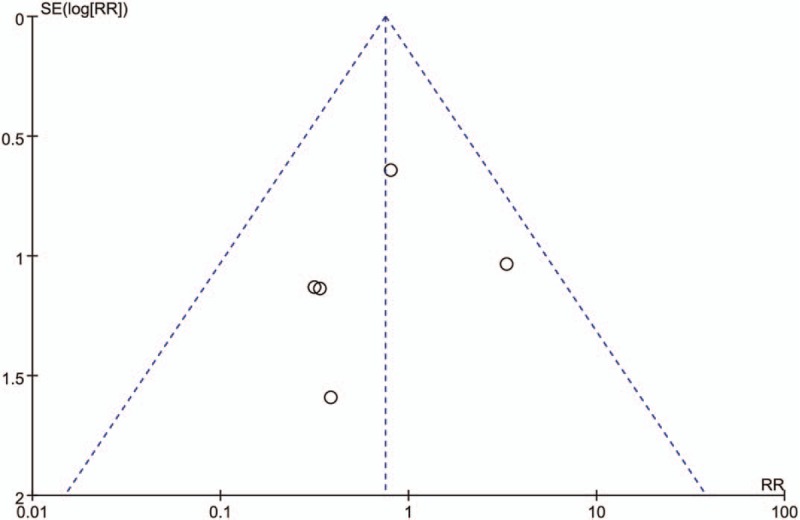
Funnel plot.

We did not assess publication bias because of the low power associated with the low number of included studies, and the potential publication bias of primary outcome was presented with funnel plot showed in Figure 5. The funnel plot of the RRs for rebleeding is basically symmetric. Therefore, we believe that the risk of publication bias is low in this meta-analysis.

In summary, these studies do not identify any differences in outcomes of patients with GIB. EEN rarely affects rebleeding and mortality, and decrease the hospitalized days in GIB patients.

## 4 Discussion

GIB is one of the most important emergency conditions despite improvement of intensive care technologies and advancements in the endoscopic treatment of GIB, mortality remains a significant problem. In our study, the all-cause mortality rate (about 10. 4%) is comparable to rates reported in other studies which amount to 10%.^[1,18]^ There are varieties of etiologies of GIB include inflammation of the digestive tract itself, mechanical damage, vascular lesions, tumor, and diseases of adjacent organs and systemic diseases can also be involved in the digestive tract. The most common cause of nonvariceal upper GIB is peptic ulcer disease,^[1,19]^ which has a wide range of rebleeding risks that can be assessed by the endoscope of recent hemorrhage. Mortality was highest in those with variceal bleeding (15%) and with malignancy (17%).^[1]^ Other etiologies for nonvariceal upper gastrointestinal hemorrhage include esophagitis, gastritis, Mallory–Weiss tears, and angiodysplasias. Endoscopic therapy is rarely required in esophagitis, gastritis, and Mallory–Weiss tears because up to 90% of these lesions stop bleeding spontaneously.^[6]^ GIB from peptic ulcers or other nonvariceal causes generally stops spontaneously; if it fails to do so, aggressive management of endoscopic therapy is required. After effective hemostasis of the initial bleeding episode, the primary concern becomes the prevention of rebleeding, which occurs in up to 20% of patients,^[3]^ so a significant portion of GIB is of low risk and rebleeding. These patients may resume enteral feeding as soon as tolerated.

Only the patients with a high risk of rebleeding should be kept nil and be hospitalized for at least 72 hours after endoscopic treatment. Most high-risk lesions become low-risk lesions within 72 hours, and most rebleeding occurs within this time.^[20]^ As a result, patients identified to be at high risk for death may be prioritized for blood transfusions and hospital admissions after GIB, and prolonged fasting may be justified. However, prolonged postponement of enteral nutrition is unnecessary or even harmful because of increased risk of stress ulceration. Importantly, there is no evidence that fine-bore nasogastric tubes cause variceal bleeding.

One retrospective study in burns^[21]^ shows that EEN may prevent the GIB (OR: 0.38; 95% CI: 0.17–0.84). Several reviews^[22–24]^ and 1 meta-analysis^[25]^ suggested that enteral nutrition may be as protection against stress ulceration and GIB. Numerous basic science studies suggest that enteral nutrition can improve mucosal blood flow and reverse the generation of these inflammatory mediators.^[22]^ The results of 1 meta-analysis suggest that, in those patients receiving EEN, stress ulcer prophylaxis may not be required.^[25]^ Consequently, enteral nutrition has multiple potential benefits and has been studied in several intensive care unit patient groups. These physiological effects may accelerate ulcer healing and reduce complications in these patients. A systematic review suggested the potential benefits of enteral nutrition include local nutrition to gastric tissue; stimulation of mucus and bicarbonate secretion by mucus glands and epithelial cells to maintain the mucus barrier; and increased splanchnic blood flow, which may facilitate ulcer healing.^[20]^ A meta-analysis attests to the feasibility of EEN in high-risk surgical patients and that these patients have reduced septic morbidity rates compared with that administered parenteral nutrition.^[26]^ Early feeding after elective open colorectal resections is successfully tolerated by the majority of patients, leading to earlier resolution of ileus and hospital discharge.^[27]^ A study indicated that EEN resulted in significant lowering of the level of proinflammatory cytokines, earlier restoration of gastrointestinal function, a decrease of complications such as infection, and shortening of hospital day in patients with severe acute pancreatitis.^[28]^

Several animal experiments support these observations in human studies.^[29–32]^ These studies use animal models have proved that enteral glucose may prevent the gastric mucosal injury associated with cold restraint stress,^[31]^ produce the increased blood flow to the terminal ileum.^[30]^ Intragastric glucose increased residual volume and gastric pH, as well as decreased gastric mucosal injury.^[32]^

These patients who are fasting also face water and electrolyte imbalances due to lack of oral feeding which decreases intestinal mucosa and causes atrophy of the intestinal wall. Then gastrointestinal septicemia may develop due to the entrance of intestinal bacteria through the atrophic intestinal wall.^[33–35]^ Besides, no correlation between rebleeding and oral feeding in patients who have GIB has been determined.

Numerous prediction models identified pre-endoscopic and endoscopic risk factors for adverse clinical outcomes in patients with GIB. The risk-stratification systems for patients with GIB discriminate between patients at high or low risks of dying or rebleeding. However, many of these predictive tools depend on endoscopic results and are, therefore, not ideal for early evaluation of patients. Several risk scores can be applied prior to endoscopy results. Among them, the most notable scales are the AIMS65 score and the Glasgow–Blatchford score, which is a simple, accurate risk score that predicts in-hospital mortality, length of stay, and cost in patients with acute upper GIB.^[36,37]^ The Glasgow–Blatchford score was equivalent in predicting the need for endoscopic therapy, rebleeding, and death and Patients with Glasgow–Blatchford scores ≤3 did not require intervention.^[36]^ A highly of patients presenting with low endoscopic risk (patients with a Glasgow–Blatchford score of 0–3) is likely to have a low risk of adverse outcomes, including rebleeding.^[36,38]^ So we could use the risk-stratification systems to decide when to initiate enteral nutrition.

Some limitations of our study need to be discussed. First, the included trials were diverse with respect to disease severity and use of nutrition therapy protocols. And the hemostatic therapy may be of some subtle differences. However, no matter how severe the disease is, the effect of the hemostatic therapy is similar in our included trails. And we strengthened the stability and accuracy of our meta-analysis by using strict trial identification, data extraction. Second, the number of RCTs fulfilling the included criterion is not enough. Only 5 trials have been included in the present meta-analysis and the sample size is small. On the other hand, the limited number of studies included in quantitative synthesis highlights the important methodological limitations in this research area. Finally, at least another large confirmatory trial is probably required to provide definite conclusions and recommendations on this issue.

In conclusion, our results indicated that EEN within 24 hours does not relate to higher rebleeding and mortality compared with DEN for patients with GIB, but decrease hospitalized days. EEN should be recommended as the preferred nutrition routine in the patients who are at low risk for rebleeding. However, multicenter, randomized clinical trials are warranted to verify these findings.

## Author contributions

**Conceptualization:** Hongyan Zhang.

**Data curation:** Guangjie Tu.

**Formal analysis:** Shujun Sun.

**Funding acquisition:** Haifa Xia.

**Investigation:** Guangjie Tu, Jingxu Wang.

**Methodology:** Yu Wang, Shujun Sun.

**Resources:** Yu Wang.

**Software:** Yu Wang.

**Supervision:** Yin Yuan, Shanglong Yao, Haifa Xia.

**Writing – original draft:** Hongyan Zhang, Yu Wang, Xin Huang.

**Writing – review and editing:** Yun Lin.
